# Granulosis Rubra Nasi Response to Topical Tacrolimus

**DOI:** 10.1155/2017/2519814

**Published:** 2017-11-29

**Authors:** Farhana Tahseen Taj, Divya Vupperla, Prarthana B. Desai

**Affiliations:** ^1^Department of Dermatology, Venereology & Leprosy, Jawaharlal Nehru Medical College and KLE's Dr. Prabhakar Kore Hospital and Medical Research Centre, Belgaum, Karnataka 590 010, India; ^2^Department of Dermatology, Venereology & Leprosy, Government District Headquarters Hospital, Khammam, Andhra Pradesh 507 002, India

## Abstract

Granulosis Rubra Nasi (GRN) is a rare disorder of the eccrine glands. It is clinically characterized by hyperhidrosis of the central part of the face, most commonly on the tip of the nose, followed by appearance of diffuse erythema over the nose, cheeks, chin, and upper lip. It is commonly seen in childhood but it can present in adults. Here we report a case of GRN in an adult patient with very unusual histopathological presentation.

## 1. Introduction

Granulosis Rubra Nasi (GRN) is also known as “Acne papulo-rosacea of the nose.”. In 1901, a German dermatologist, Jadassohn, had described the first case of GRN as “Nasi hyperhidrotic Erythematosa micropapules Dermatosis Infantum” [1]. It is an inflammatory dermatosis involving eccrine sweat glands of central face and clinically presents with hyperhidrosis, erythema, papules, pustules, and vesicles. Rarely small comedo like lesions may be present [2].

It is usually limited to the front and sides of the nose. It may also affect the eyebrow, upper lip, and cheek. Presentation is common in childhood with a peak age of presentation at 7–12 years, but adolescent and adult onset is also possible. It has a chronic course and resolves at puberty without any sequelae. It is described as a focal form of hyperhidrosis which differs from the other forms, as it does not depend on the hypothalamic or emotional stimuli [3].

## 2. Case Report

**Figure 1:**
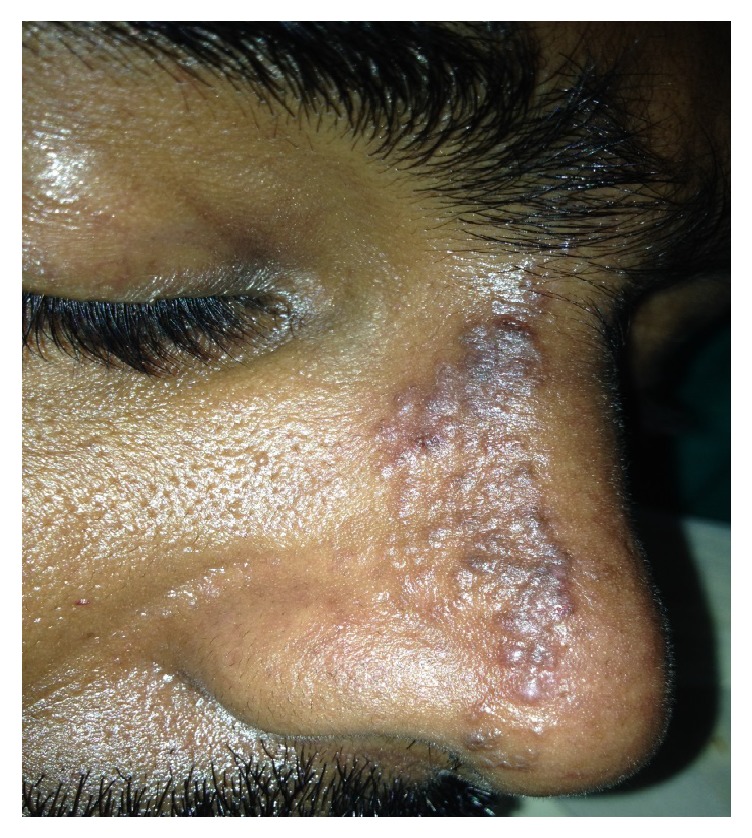
Multiple hyperpigmented papules and some vesicles present over the right side of the nose.

A 33-year-old male patient presented with asymptomatic lesion over nose since 2 years to the outpatient department of dermatology and leprosy. The patient gave no history of any treatment taken before. There was no history of any fluid or cheesy material coming out of the lesions and no history of itching or burning (Figure 1). Clinically, we made a diagnosis of Granulosis Rubra Nasi, Lymphangioma Circumscriptum, Nevus Comedonicus, and sebaceous gland hyperplasia.

**Figure 2:**
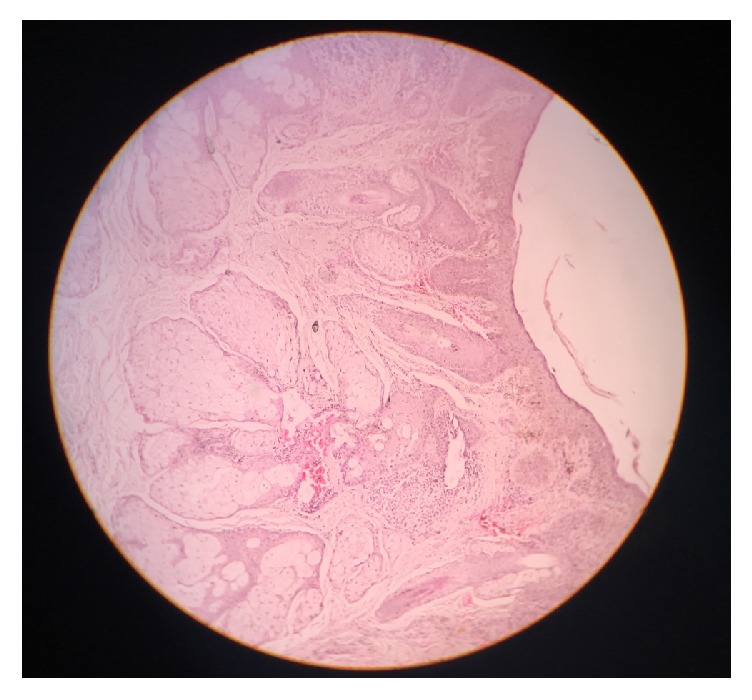
Histopathology: epidermal hyperplasia with spongiosis. Dermis shows villous hair follicle with sebaceous gland hyperplasia with peri-infundibular infiltrate of lymphocytes and plasma cells with dilated capillaries and extravasation of hemosiderin.

A biopsy of the skin lesion was done. The histopathology report showed epidermal hyperplasia with spongiosis. Dermis shows dilated eccrine sweat glands. The infundibular and sebaceous ducts are plugged with stratum corneum and villous hair follicles. There is moderately peri-infundibular infiltrate of lymphocytes and plasma cells. Papillary dermis shows dilated capillaries with extravasation of hemosiderin (Figure 2).

**Figure 3:**
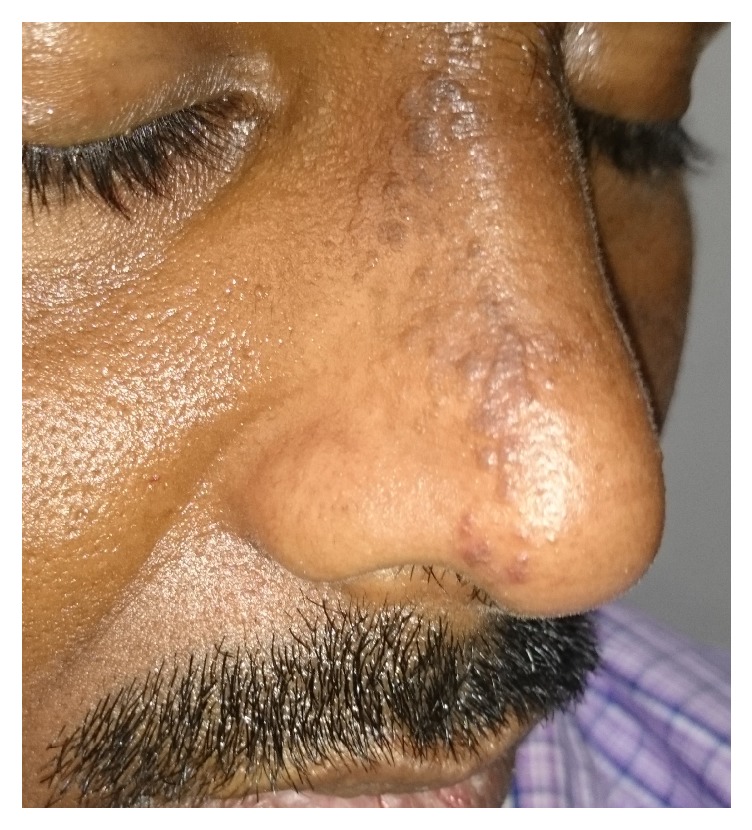
Three weeks after treatment with topical tacrolimus.

The patient has been treated with topical tacrolimus 0.03% and systemic corticosteroids. After 15 days of follow-up, the patient has shown good response (Figure 3).

## 3. Discussion

This is a chronic, benign condition of unknown etiology [2, 4]. It is rare with autosomal dominant or autosomal recessive pattern of inheritance. The gene locus remains unidentified [5]. Persistent localized hyperhidrosis of central face is the main cause for this condition [6]. GRN usually starts in early childhood and resolves spontaneously at puberty, but rarely it may persist [7] Males are most commonly affected. 6 out of 7 patients described by Jadassohn were boys [1]. This condition is usually asymptomatic except for mild pruritus.

Hyperhidrosis is the initial conspicuous feature of GRN which tends to worsen in summer. Excessive sweating may precede other changes by several years. Small beads of sweat can be seen at the tip of the nose. As a result of persistent hyperhidrosis, diffuse erythema develops over the tip of the nose. Erythema may gradually extend to involve upper lip, cheeks, and chin with sweat droplets studded over, giving glistening appearance. Small erythematous macules, papules, vesicles, or pustules lesions can also be seen [2, 8]. These lesions disappear on diascopy and reappear on relieving pressure [2].

The pathogenesis is unknown. It is an inflammatory dermatosis involving eccrine sweat glands of central face involving nose, cheeks, or chin, representing a unique sweat retention form [2]. Some authors have suggested a defect in vasomotor and secretory functions of the nose. Presence of significant increase in sweating on the nose and central face appears to be responsible for the secondary changes like erythema and erythematous papules [9].

The differential diagnosis like rosacea or perioral dermatitis can be considered. But, in rosacea, there is erythema over nose and cheeks along with telangiectasias but there is no hyperhidrosis of the central part of the face. Perioral dermatitis can present with erythema, small monomorphic papules, and pustules with or without scaling involving the perioral area without hyperhidrosis. Other differential diagnoses include acne vulgaris, lupus pernio, lupus erythematosus, lupus vulgaris, leishmaniasis, actinic keratosis or skin cancer, miliaria crystallina, and hidrocystoma [2, 4, 10]. Acne vulgaris presents with comedones, papules, and pustules without hyperhidrosis and telangiectasia. Lupus pernio or chilblain lupus presents with dusky papules and plaques on the nose, toes, and fingers. Lupus erythematosus has history of photosensitivity. In acute lupus erythematosus, there is a malar rash with mouth ulcers but there is no hyperhidrosis. Miliaria crystallina presents with vesicles mainly over the areas occluded by clothes.

Association with primary palmoplantar hyperhidrosis, acrocyanosis, and poor peripheral circulation was observed. Kumar et al. had reported an association with another eccrine gland disorder, hidrocystoma [8]. Heid et al. had reported an association with rhinorrhea [11]. Heid et al. reported pheochromocytoma with GRN in a 19-year-old woman who showed regression of hyperhidrosis and the nasal dermatosis after surgical removal of the tumor [11]. Barber had suggested involvement of adenoids, which can provide a source of irritation at the tip of the nose [12]. Topical indomethacin, drying lotions like calamine, tetracycline, cryotherapy, and X-rays (temporary benefit) have been described in the treatment of GRN [13]. Recently, use of botulinum toxin A that induced long-term remission in a patient with GRN was described by Grazziotin et al. [14]; botulinum toxin A improves GRN by decreasing hyperhidrosis.

Granulosis Rubra Nasi presents with three stages: initial hyperhidrosis followed by erythema and papular lesions and late vesicular stage. Our patient presented with late vesicular stage, so topical tacrolimus 0.03% was advised with good clinical response. There are no complications associated with the condition and the disease has excellent prognosis with self-resolution. Systemic corticosteroids help by reducing the inflammatory infiltrate around sweat glands. Topical tacrolimus has been used in low dose, 0.03%, with excellent response [8].

The diagnosis is usually clinical. Histology shows dilation of dermal blood vessels and lymphatics with perivascular lymphocytic infiltration and dilation of sweat ducts. Eccrine hidrocystoma also shows dilatation of sweat glands with solitary or multiple cysts lined by a double layer of cuboidal cells on histology, but dilatation of dermal vessels and perivascular mononuclear infiltrate is not seen [4, 10].

## 4. Conclusion

GRN is a rare disorder. One should remember that it could be a complication of hyperhidrosis. Treatment is symptomatic and cosmetic. Counseling the patients about the self-limiting nature of the condition is of paramount importance. To the best of our knowledge, there are not any case reports showing GRN with sebaceous gland hyperplasia.
